# The Muscle Cells in Pelvic Floor Dysfunctions: Systematic Review

**DOI:** 10.3390/muscles4010009

**Published:** 2025-03-18

**Authors:** Ana Margarida Vieira, Maria Leonor Faleiro, Miguel Mascarenhas-Saraiva, Sandra Pais

**Affiliations:** 1 Unidade de Portimão de Gastrenterologia, Unidade Local de Saúde do Algarve, 8500-338 Portimão, Portugal; 2 Faculdade de Medicina e Ciências Biomédicas, Universidade do Algarve, 8005-139 Faro, Portugal; 3 Faculdade de Ciências e Tecnologia, Universidade do Algarve, Campus de Gambelas, 8005-139 Faro, Portugal; mfaleiro@ualg.pt; 4 ManopH-Laboratório de Endoscopia e Motilidade Digestiva, Lda, 4000-432 Porto, Portugal; miguelms.manoph@gmail.com; 5 Comprehensive Health Research Center, 7004-516 Évora, Portugal; sandra.rafael.pais@gmail.com

**Keywords:** pelvic floor dysfunction, pelvic floor muscles, muscle, extracellular matrix, pelvic floor muscle function

## Abstract

Background/Aims: The pelvic floor muscles are important structures involved in pelvic floor tone, pelvic organ support, and continence. The aim of this study was to perform an update on the pelvic floor muscle structure and function alterations of women with pelvic floor dysfunctions. Methods: A systematic search was undertaken in two electronic databases, PubMed/Medline and Ovid Discovery to find manuscripts (in English), published between 1 January 2002 and 31 July 2022, including all clinical studies using the following search terms: “muscle” or “extracellular matrix *” and “pelvic floor dysfunction *”. All clinical trials, observational, or animal studies examining the muscle and reporting pelvic floor dysfunction as a primary outcome were included. Case reports, literature reviews, conference papers and theses, and unpublished data were excluded. To ensure that no eligible articles were overlooked, the reference lists of all included papers underwent manual scrutiny. The bias level was estimated using Newcastle–Ottawa Scale (NOS) for cohort and case-control studies. A qualitative synthesis was performed. Results: The significant qualitative and quantitative heterogeneity between the studies did not allow for a quantitative analysis. Of the 30 articles selected with a total of 5592 women, 15 referred to the analysis of structural muscle defects, which included 3365 participants with urinary incontinence, pelvic organ prolapse, fecal incontinence, cistocele, rectocele, and sexual dysfunction; 10 manuscripts referred to the study of pelvic floor muscle function with a population of 2042 women, such as urinary incontinence, pelvic organ prolapse, fecal incontinence, and sexual dysfunction; and 5 papers evaluated cellular and/or molecular changes affecting the pelvic floor muscles, like urinary incontinence, pelvic organ prolapse, and rectocele, which included a total of 185 participants. Women with pelvic floor muscle defects are at greater risk of pelvic floor dysfunctions, and inversely, women with pelvic floor dysfunctions have more pelvic floor muscle defects than women without pelvic floor dysfunctions. These patients demonstrate a reduction in muscle tone, contraction strength, and resistance, a compromised neuromuscular activity, and an alteration of the normal composition and organization of the muscle cells. Conclusions: Women with pelvic floor dysfunction have anatomical muscle defects, disturbance of muscle function and cellular changes involving muscle cells and nerve fibers.

## 1. Introduction

Pelvic floor disorders include urinary incontinence, pelvic organ prolapse, fecal incontinence, and other sensory and emptying abnormalities of the lower urinary and gastrointestinal tracts [1]. Pelvic floor dysfunction (PFD) is a prevalent condition affecting approximately 25% of the community-dwelling women in low- and middle-income countries [2]. In the United States, population-based studies’ results are similar, nearly one-quarter of all women reported symptoms of, at least, one pelvic floor disorder [1,3]. The prevalence of pelvic floor disorders significantly increases with age: 40% of women aged 60–79 and 53% of women aged higher 80 suffer from, at least, one symptomatic disorder [3]. With the growing population of older women, the nationwide impact of pelvic floor disorders, in terms of healthcare expenses, diminished productivity, and reduced quality of life, is anticipated to be significant [1].

The pelvic floor structures include the muscular tissues in the pelvic floor and their neural connections, and the fascial (connective tissue) layers surrounding the pelvic floor muscle fibers/fascicles composing and working like a neuro-mio-fascial functional unit [4]. The muscles, at rest, are tonically and simultaneously contracted, in order to provide pelvic floor tone, support the pelvic organs, and maintain continence [5].

The pelvic floor muscles are one of the central pillars that contribute to PFD, so it is of great importance to be aware of the different muscle changes, the way in which they interact with each other and the respective functional consequences, in order to better understand the underlying mechanisms of PFD.

The current review aimed to provide an overview of the functional muscle alterations and/or macro and microstructural alterations observed in PFD.

## 2. Materials and Methods

### 2.1. Design

#### Database Search and Study Selection

This manuscript was designed using the Preferred Reporting Items for Systematic Reviews and Meta-Analyses (PRISMA) guideline [6].

A systematic search in two electronic databases, PubMed/Medline and Ovid Discovery, was conducted to identify relevant publications (in English) released between 1 January 2002 and 31 July 2022. The established protocol was registered at the International Prospective Register of Systematic Reviews (PROSPERO) CRD42023395631.

### 2.2. Eligibility Criteria

Between 1 January 2002 and 31 July 2022, all publications were selected according to the following criteria: (i) either clinical trials, observational, or animal studies, (ii) examining the muscle, and (iii) reporting pelvic floor dysfunction as a primary outcome. Case reports, literature reviews, conference papers and theses, and unpublished data were excluded from the study. The entire reference list of all the included articles was manually evaluated, thus avoiding the loss of relevant articles.

### 2.3. Search Strategies

The principal investigator researched clinical studies using combinations of the following search terms: “muscle” or “extracellular matrix *” and “pelvic floor dysfunction *”. The databases consulted were PUBMED (The US National Library of Medicine) and OvidDs (Ovid Discovery).

### 2.4. Study Selection and Data Extraction

A total of 857 articles were selected by title: 293 from PUBMED and 564 from OvidDs, published over the last 20 years. Duplicate articles were excluded from the analysis (n = 43). The remaining 814 articles underwent evaluation by two investigators to reassess the titles and review the abstracts. Articles meeting the inclusion criteria (clinical studies) were selected, while those meeting the exclusion criteria (studies exclusively evaluating extracellular matrix, case reports, literature reviews, conference papers and theses) were rejected. Subsequently, 36 articles were chosen for comprehensive examination of their full texts. Any disagreements between the two investigators were resolved by the other two investigators. At this stage, 21 out of the 36 articles were excluded from both qualitative and quantitative synthesis for the following reasons: all the patients had no pelvic floor dysfunction (n = 9), different objectives and study designs (n = 7), exclusive assessment of the extracellular matrix (n = 4); study without muscle evaluation (n = 1). The 15 manuscripts not excluded were added to the 15 articles selected from the reference list of the included publications obtaining 30 articles that were included in the final analysis.

**Figure 1:**
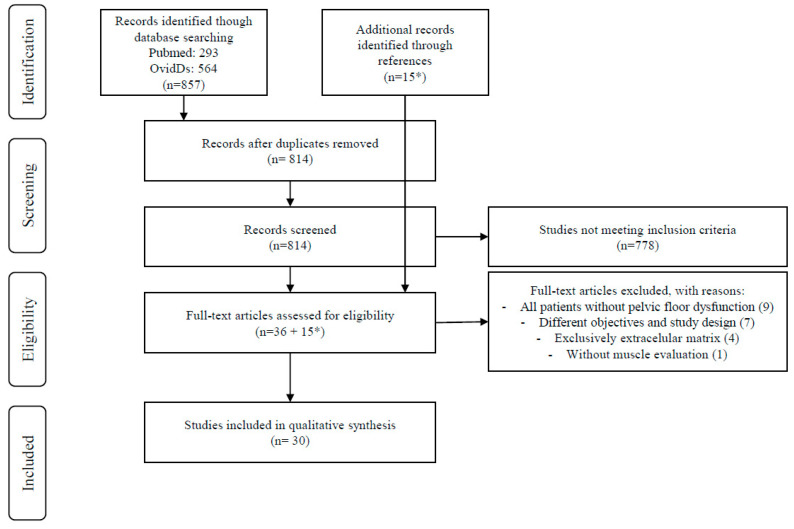
Study selection process—PRISMA flow diagram.

The various stages of information flow in the review are depicted in Figure 1 (PRISMA).

### 2.5. Risk-of-Bias Assessment and Data Synthesis

A qualitative synthesis is presented, which reviews all the pelvic floor muscle changes found in pelvic floor dysfunctions and summarizes the role of the muscles in these disorders.

The bias level was estimated using the Newcastle–Ottawa Scale (NOS) for cohort and case-control studies [7].

## 3. Results

### 3.1. Study Characteristics

Of the 30 articles selected with 5592 participants, 15 reported the analysis of structural muscle defects, 10 manuscripts referred to the study of pelvic floor muscle function, and 5 articles evaluated the cellular and/or molecular changes affecting pelvic floor muscles.

Although it was not an inclusion criterion, all the selected assessed studies were only women.

The design of the studies was divided between cohort studies and case-control studies.

Significant qualitative and quantitative heterogeneity was observed among the studies, even within each subgroup with similar primary outcomes. This heterogeneity refers to different studied populations, parameters, and measurement methods. For this reason, it was not possible to carry out a quantitative analysis.

The characteristics, and demographic and clinical data of the included studies are listed in Tables S1 and S2 in the Supplementary Materials.

### 3.2. Study Quality Appraisal and Bias Assessment

The quality of the studies was globally good, but most of them had some bias selection risk (Table S3, Figures S1 and S2 in the Supplementary Materials). According to this evaluation and due to the studies’ heterogeneity, the results could not be entirely representative of the entire population.

#### 3.2.1. Cellular Pelvic Floor Muscle Alterations

The authors selected five articles that studied cellular and/or molecular PFM [8,9,10,11,12]. All the studies were case-control studies. They included a total of 185 participants with different pelvic floor dysfunctions (UI, POP, and rectocele) and distinct primary outcomes, such as, peripheral nerve abnormalities, rectovaginal innervation, genital sensory and motor enervation, *muscularis propria* changes of the anterior vaginal wall, and molecular expression of the vaginal anterior wall (Table S2).

Patients with POP have a decreased expression of the adenosine diphosphate ribosylation factor GTPase activating protein 3 (ArfGAP3) gene and its expression is negatively correlated with POP severity [8]. ArfGAP3 downregulation affects cytoskeleton-related molecular motors, such as actin and myosin, and Ca^2+^ vesicle transport [8]. Furthermore, the imbalance of Ca^2+^ homeostasis causes the abnormal metabolism of peptide neurotransmitters and extracellular matrix [8].

Busacchi et al., 2004 [9] demonstrated that in muscle specimens from women with genitourinary prolapse, both the density and intensity of neuropeptide Y, vasoactive intestinal polypeptide, and substance P immunoreactive nerves were significantly decreased compared to control specimens [9]. Therefore, the decrease in neurochemical levels detected within the neural network supplying this region might play a role in changing the functionality of the perineal muscles, which are implicated in stress urinary incontinence and genitourinary prolapse [9].

North et al., 2013 [10] demonstrated a higher proportion of polyphasic potentials, indicating a more pronounced degree of partial denervation (followed by subsequent reinnervation) of the left pubococcygeus muscle compared to the women in the control group. This finding aligns with a decline in motor function of the pelvic floor nerves in patients with POP [10].

Altman et al., 2006 [11] discovered a significant association between increasing perineal descent via defecography and nerve fiber immunofluorescence intensity, using protein gene product (PGP-9.5) antibodies (OR 1.3, 95% CI 1.1–2.1), although not for the size of the rectocele (OR 0.5, 95% CI 0.9–1.2) or rectocele contrast medium emptying at defecation (OR 0.9, 95% CI 0.8–1.1) [11]. The PGP-9.5 neuronal marker does not discriminate between motor or sensory nerve fibers. Considering that the nerve fibers were identified in this investigation as free nerve endings, dispersed within the deeper layers of the rectovaginal wall, it is likely that they were mostly of a sensory origin [11].

The analysis of prolapsed fragments from the anterior vaginal wall using Masson’s Trichrome staining revealed evident disarray within the muscular layer, characterized by a distorted muscle tissue architecture with substantial collagen accumulation within smooth muscle cells, together with a reduction in elastic fibers. In the control samples, smooth muscle cells (SMCs) exhibited a denser arrangement, organized in aligned fibers, with a more uniform distribution of elastic fibers. Immuno histochemistry and immunofluorescence assessments revealed a significant elevation in Platelet-derived Growth Factor (PDGF) expression within the *muscularis propria* of prolapsed specimens, contrasting with controls where immunostaining was either mild or absent. In the control fragments, the α-smooth muscle actin (α-SMA) immunoreactivity of SMCs was confined to customary locations, displaying a consistent distribution within the longitudinal and circular muscle layers of the *muscularis propria*. However, in the prolapsed fragments, α-SMA expression exhibited disarray among smooth muscle cells, seemingly displaced by heightened connective tissue presence, consequently altering the architectural arrangement of the *muscularis propria* [12].

#### 3.2.2. Structural Pelvic Floor Muscle Changes

We selected 15 articles reporting structural PFM changes [13,14,15,16,17,18,19,20,21,22,23,24,25,26,27]. Most of the studies were case-control studies [13,14,16,17,19,20,22,24,25,26], and only five were cohort studies [15,18,21,23,27]. We included 3365 women with UI, FI, POP, cystocele, rectocele, and sexual dysfunction. With different tools and parameters, LAM, pubovisceral, puborectalis, and anal sphincters were studied.

Most of the studies proved that muscle injuries increase the risk of pelvic floor dysfunctions, but with different impacts on the various pelvic compartments.

Among women with sphincter tears, those with concomitant major *levator ani* muscle (LAM) injuries showed trends towards more fecal incontinence symptoms and pelvic organ prolapse [13].

Van Delft et al., 2014 [14] found, in women with major LAM avulsion, more postnatal urinary incontinence, but no difference was found for fecal incontinence or prolapse symptoms [14]. Another prospective study showed that the depth and width of levator ani defects were associated with the likelihood of symptoms of prolapse and cystocele descent, but not with urinary incontinence [15]. No correlation was found between LAM injury and pelvic floor muscle contraction with anal incontinence or urinary incontinence symptoms, at a median follow-up of 5 months, after sustaining an obstetric anal sphincter injury (OASI) [16].

Women with a prior episiotomy or anterior vaginal wall reconstructive surgery had a higher probability of experiencing more severe pubovisceral muscle avulsions (adjusted OR, 3.77 and 3.29, respectively). Similarly, women exhibiting symptoms of POP had increased odds (OR, 1.01 per unit increase) of encountering more severe avulsions, as did those with higher-stage POP of the central vaginal compartment, as measured by POP-Q “C” (OR, 1.18). Conversely, women with symptoms of obstructive defecation were less likely to exhibit defects in the pubovisceral muscle on Magnetic Resonance Imaging (MRI) (OR, 0.97 per unit increase) [17].

Prolapse was seen in 150/181 (83%) women with LA defects and in 265/600 (44%) women without defects (*p* < 0.0001), resulting in a relative risk (RR) of prolapse of 1.9 (95% CI 1.7–2.1) among women with defects. The prevalence of levator avulsion was, approximately, four times greater in women with significant clinically diagnosed pelvic organ prolapse (POP-Q stage II or higher) compared to those without prolapse. The association was most pronounced in the anterior and central compartments, with a RR of 2.3 (95% CI 2.0–2.7) for cystocele and 4.0 (95% CI 2.5–6.5) for uterine prolapse. Furthermore, women with bilateral avulsion were notably more likely to experience uterine prolapse, with a RR of 7.1 [18].

Primiparas, with stress urinary incontinence (SUI) have twice the chance of having a muscle abnormality, when compared with continent women [19]. The authors also found that LAM injury, not only predominantly affects the pubovisceral portion of the LAM, that arise from the inner surface of the pubic bone just lateral to the vagina, but it also involves the iliococcygeal muscle [19]. The analysis of the current patient population revealed that 71% of women with late-onset fecal incontinence (FI) after vaginal delivery had an anatomical sphincter defect [20]. However, Dietz et al., 2009 [21] confirmed a highly significant negative relationship (*p* < 0.001) between stress urinary incontinence and levator trauma, in women over the age of 50, while there was no significant relationship in younger women. The analysis of urge urinary incontinence confirmed a significant negative relationship between urodynamic stress incontinence and levator trauma, after adjusting for other significant risk factors (*p* = 0.002) [21]. Levator avulsion showed a significant association with prolapse beyond the hymen (odds ratio, 2.7; 95% confidence interval, 1.3–5.7), as well as with the symptoms of prolapse (odds ratio, 3.0; 95% confidence interval, 1.2–7.3) [22]. Even after accounting for forceps-assisted delivery, these associations remained consistent.

Discovered via MRI, women with SUI presented a reduced urethral sphincter muscle thickness of its posterior portion (37%), an omega shape (13%), or higher signal intensity (50%); its abnormal configuration was associated with an increased signal intensity of 70% (*p* = 0.001) [23]. The LAM exhibited unilateral loss of substance in 30%, a higher signal intensity in 28%, and an altered origin in 19%. Central defects of the endopelvic fascia were present in 39% (n = 21) and lateral defects were present in 46% [23]. There was a significant association between the loss of the symphyseal concavity of the anterior vaginal wall and lateral fascial defects (*p* = 0.001) and levator ani changes (*p* = 0.016) [23]. The study of Luo Y. et al., 2020 [24] of 44 women to investigate how a unilateral high-grade tear caused the overdistension of the LAM and whether the tear positions affect the degree of distension, reported 42% of SUI and 11% of prolapse symptoms in the 26 postpartum women with unilateral *levator ani* defect (LAD) group, unlike the 18 nulliparous women with an intact LAM group that did not complain of any symptoms [24]. The valsalva anteroposterior diameter (AP), rest and valsalva coronal diameter (LR), and rest and valsalva hiatal area (HA) of the levator hiatus in women with a unilateral high-grade LAD were larger than those in women with an intact LAM (*p* < 0.05) [24]. The difference between valsalva and rest of AP, LR, and HA in women with a unilateral high-grade LAD were significantly larger than those in women with an intact LAM (*p* < 0.05), and these values were higher if the tear was in a cephalad position instead of a caudad tear [24]. The levator attachment width (LAW) measurements were significantly higher in women who had been diagnosed with no defect (*p* < 0.001): 13.83 ± 2.99 mm with an intact LAM versus 8.95 ± 3.44 mm with a unilateral high-grade tear [24].

In a population with FI, the utilization of 3D endovaginal and anorectal ultrasonography revealed a significant association between pubovisceral muscle (PVM) defects and an increased laterolateral diameter and levator hiatus area, both at rest and during a Valsalva maneuver [25]. There was a significant positive correlation between the fecal incontinence score and ultrasound score, encompassing anal sphincters and PVM defects (*r* = 0.63, *p* = 0.01). Conversely, no significant correlations were observed between the incontinence scores and the levator hiatal area (r = 0.43, *p* = 0.76), nor between the ultrasound scores and the levator hiatal area (*r* = 0.24, *p* = 0.08) [25].

With the utilization of 3D MRI, it was observed that women with major defects exhibited a 36% reduction in the cross-sectional area in the ventral portion of the LAM, compared to women without defects. In an opposite way, in the posterior portion of the muscle, women with major defects displayed a 9% larger cross-sectional area, compared to those without defects, but for each defect severity category (none, minor, major), there were no significant differences in the cross-sectional area, between women with and those without prolapse [26].

The data obtained from the pre-perineal rehabilitation, magnetic resonance examination in 15 patients with a SUI diagnosis showed an asymmetry of the LAM in 87% of the patients and a bilateral volume reduction of LAM in the remaining 13%. Within these patients, the muscle was symmetrically replaced by fibro-fatty tissue [27]. Patients with a complete response to rehabilitation showed an increase in muscular trophism, with resolution of the asymmetry between the muscles of the two sides and an increase of 39% in the cross-sectional area [27]. In the partial response group (20%), there was a more consistent gain in tropism observed on the side that exhibited less trophic activity before therapy, resulting in an overall increase in trophism. The cross-sectional area of the muscle increased by about 29% with a greater proportion of the hypotrophic section (increase of 47%). However, there remained a significant disproportion between the muscles on the two sides: the healthy muscle still exhibited a 42% larger size compared to the muscle on the other side [27]. Analyzing the examinations of non-responder patients, it was noted that the perineal rehabilitation was ineffective when also considering the gain in tropism. The cross-sectional area of the muscle remained unchanged and low. The overall increase in the cross-sectional area was about 10% [27].

#### 3.2.3. Muscle Function

We described the 10 articles based on PFM function studies of patients with PFD [28,29,30,31,32,33,34,35,36,37]. Only three studies were cohort studies [32,34,36], and all the other studies were case-control studies [28,29,30,31,33,35,37]. These studies included 2042 participants. Applying different methods, the PFM function was analyzed in UI, POP, FI, and sexual dysfunction.

Healthy women exhibited higher levels of pelvic floor muscle (PFM) tone, maximum strength, neuromuscular activity, and resistance compared to both puerperal mothers and women with pelvic floor disorders (*p* < 0.01). Puerperal women and those with pelvic floor disorders (including UI, FI, POP) demonstrated similar functional PFM values [28].

Women with SUI showed reduced levels in passive force, absolute endurance, maximum speed of force generation, and the total number of muscle contractions, when compared with continent women [29]. In the controlled study involving women with POP, it was found that women with prolapse had a lower adjusted maximal contraction (2.0 N) than the controls (3.2 N, *p* < 0.001) [30].

Tosun G et al., 2019 [31] assessed the PFM function via digital palpation using the PERFECT scale of 82 midwives and nurses of reproductive age with (n = 51) and without PFD (n = 31). The power parameters of the PERFECT scheme representing the strength of the PFMs were significantly lower in subjects with PFD (POP and UI) compared to the non-PFD group (*p* = 0.002). The PFM strength was negatively correlated with urinary incontinence symptoms and with the number of PFD (*p* = 0.002, *r* = −0.34) [31]. In another study, women with different stages of prolapse (n = 317, 13% had stage II prolapse, 68% stage III, and 19% stage IV) were compared [32]. Those with stage II prolapse had a higher Brink score (*p* = 0.04), primarily due to the vertical displacement subscale (*p* = 0.03). This suggests that women with a less advanced stage of prolapse were able to better elevate their pelvic floor muscles during contraction, compared to those with more severe prolapse. Women in the lower Brink scale quartile had larger genital hiatus measurements when straining and more urinary symptom burden [32].

Prolapse was identified in 109/429 (25%) of vaginally parous women and was significantly associated with levator avulsion (odds ratio (OR) 4.17, 95% confidence interval (CI) 2.28, 7.31) [33]. Prolapse was also associated with the levator hiatus area (OR 1.52 per 5 cm^2^, 95% CI 1.34, 1.73) and inversely with muscle strength (OR 0.87 per 5 cm H_2_O, 95% CI 0.81, 0.94) [33]. In a multivariable logistic model including levator avulsion, levator hiatus area, and strength, the association between levator avulsion and prolapse was no longer statistically significant (OR 1.75, 95%CI 0.91, 3.39). The hiatus area and strength mediated 61% (95% CI 34%, 106%) of the association between avulsion and prolapse, and probably the two markers can explain this association [33].

In this study [34], the authors analyzed the functional changes of PFM between three different groups (healthy women vs. postpartum women vs. women with PFD). Women with pelvic floor disorders and postpartum women presented statistically significant lower values of basal tone, maximum strength, neuromuscular activity, and stretching resistance compared with healthy women [34].

Women with urinary continence at mid-pregnancy and at 6 weeks postpartum had a significantly higher PFM strength and endurance than their incontinent counterparts (*p* < 0.05) [35].

In a cohort of women with FI, in addition to the lack of association with puborectalis avulsion, or the hiatal area at rest and on maximum Valsalva, there was no association between the surrogate measures of the biomechanical properties of the puborectalis muscle (muscle fiber strain) on Valsalva (*p* = 0.10) or pelvic floor muscle contraction (*p* = 0.693) and FI, or any of the other symptoms of anal incontinence [36].

LA defects are significantly more common in fecally incontinent older women and are strongly associated with FI, even when adjusting for defects in the external anal sphincter [37]. Additionally, older women experiencing FI demonstrate an inability to increase their pelvic floor strength. This is evident through direct measurement, which reveals a decreased contractile force of the levator ani muscles during instrumented speculum examination, as well as indirect measurement, indicating the inability to elevate the perineal structures on MRI [37].

## 4. Discussion

The objective of this study was to perform an update on the PFM structure and function alterations in patients with PFD. This is the first systematic review to compile all the muscle alterations found in people with pelvic floor dysfunctions, from molecular to functional changes, aiming to establish a theoretical relationship between them and improve the understanding of the pathophysiology of these conditions.

Most of the available published data are focused on POP followed by UI. Regarding posterior compartment dysfunctions, like FI, the available information is very limited. Data are more robust and reproducible in respect to morphological muscle changes found in patients with PFD, independently of the affected compartment. The same is not true for the cellular alterations and muscle function data, which present few and controversial data and mainly refer to patients with POP. The investigation of the function of the pelvic floor muscles is extremely heterogeneous, firstly due to the use of different measurement tools. In the future, it will be an important progress to establish a gold standard assessment for pelvic floor muscle function. In this field, the development of pelvic electromyography and tensiomyography could prove to be important study tools for a better understanding of pelvic floor dysfunctions, as well as for the development of different and personalized therapeutic options.

It is also relevant to mention that, in all the reviewed studies, the risk of bias on selection may not be negligible, since few community studies were found, which may affect the sampling of the entire spectrum of PFD.

The studies about morphological changes have some significant limitations because they use different imaging methods, namely transperineal/translabial ultrasonography, endorectal and endovaginal ultrasonography, MRI, with different protocols and rely on methods that are highly dependent on the operator’s experience.

Nevertheless, our review proved that women with PFM defects have a higher risk of PFD and inversely, women with PFD have more PFM defects than women without PFD [13,14,15,16,17,18,19,20,22]. Some controversial or negative results [14,15,21,22] are probably due to the importance of many other factors, such as the LAM defects location. For example, the defects of LAM are more associated with PFD and involved more frequently the pubovisceral and iliococcygeal muscles [17,19]; cephalad LAM tears, instead of a caudad localization, are more associated with UI [24]; and bilateral LAM avulsion causes more uterine prolapse [18]. Other concomitant alterations may also have an influence, such as the association of LAM avulsion with anal sphincter tears, which is an important factor for FI [13,25]. The depth and width of LA defects influence the likelihood of prolapse and cystocele [15], a reduced urethral sphincter muscle thickness, an omega shape, central defects of the endopelvic fascia [23], or a reduced levator attachment width [24] seen in UI women. Recently, a study was published reporting the anatomic structure of the LA using a novel 3D digitized muscle-mapping approach based on layer-by-layer dissection [38]. The authors described the LA using a new nomenclature based on muscle bundles origin-insertion. Their work underlines the complexity of the LA and the different functions and structure of the different bundles that compose this muscle. The parts of the LA involved in the rectum are the functional portion of the muscle, which is very important for the normal urinary and defecation functions [38].

Another important aspect is the consequence of these alterations. Actually, damaged LAM integrity can result in the overdistension of the LAM [24] and muscle hypotrophy [27]. In severe cases of muscle tropism, a fibro-fatty tissue replacement and muscle denervation is recognized [27], which is in line with the results of studies that investigated the cellular alterations that characterize patients with PFD.

The Vetuschi et al., 2016 [12] study also showed that in women with genital prolapse, there were observable morphological changes in the anterior vaginal wall. These changes were characterized by a disorganized architecture of the muscularis, accompanied by a significant increase in collagen deposition within smooth muscle cells, alongside a decrease in elastic fibers [12].

According to the authors, PGDF may be involved in this trans-differentiation of metaplastic smooth muscle cells to myofibroblasts. This transition seems to lead to heightened levels of type III collagen within the muscularis layer, stemming from the phenotypic conversion of smooth muscle cells to myofibroblasts, without undergoing apoptosis [12]. This can be particularly interesting and have a greater impact, knowing that the visceral medial levator ani muscle insertions are mainly composed of smooth muscle cells. These smooth muscle fibers form a connection between the levator ani muscle and the rectum or vagina, establishing an interface [39,40]. They are accompanied by autonomic nerves from the inferior hypogastric plexus [40].

Furthermore, patients suffering from PFD show reinnervation (sign of denervation) [10] and neuropeptide depletion [9]. After motor nerve injury followed by reinnervation, each remaining fiber innervates more motor units than before the injury [10]. Therefore, the loss of one nerve fiber after reinnervation has a greater impact on function [10]. Various factors could potentially contribute to neural dysfunction, resulting in the gradual loss of neuropeptides [9]. Pelvic floor nerve fiber dysfunction may result in a generalized weakness and laxity of the affected muscles, such as the puborectalis and *levator ani* [11]. The affected nerve endings might also engage with connective tissues, blood vessels, and pelvic floor muscles through alterations in the release of growth factors and other substances that modulate tissue behavior [11]. Although the underlying mechanisms leading to neurochemical depletion remain unknown, the downregulation of the ArfGAP 3 gene could be one of the underlying mechanisms leading to the neurochemical depletion [8].

All these alterations and several still unknown changes lead to a loss of muscle function. Globally, although not knowing exactly the contribution of each structure (pelvic floor muscles, accessory muscles, tendons, conjunctive tissue, nerves), it seems that patients with PFD have a decreased contraction force and resistance. This may also be related to an impaired ability to recruit fast fibers from the PFMs, type II fibers (fast twitch), and an impairment of type I fibers (slow twitch), the most predominant fibers of the pelvic floor muscles. Another important aspect is the length of the muscle. An increase in the length of the muscle reduces its ability to contract because of the suboptimal sarcomere lengths present in the myofiber [34].

The presence of a lax perineum accompanied by a reduction in the thickness of the PFM and an enlargement of the urogenital hiatus, supports the observed decrease in muscle tone, contraction force, resistance, and neuromuscular activity in women with PFD [28].

Women with pelvic floor dysfunctions not only experience the functional impairment of the pelvic floor musculature due to lacerations or other types of muscle damage, but this is also due to molecular changes that lead to alterations in neuromotor function, muscle fiber disarray, and muscle fiber replacement. This helps to explain why individuals with a seemingly normal anatomy may still have the condition and why pathology can persist even after anatomical correction.

## 5. Conclusions

Women with PFM defects have a higher risk of PFD, and inversely, women with PFD have more PFM defects than women without PFD. Some controversial or negative results are probably due to the importance of many other factors, such as the LAM defects location.

Damaged LAM integrity can result in the overdistension of the LAM, muscle hypotrophy, a fibro-fatty tissue replacement, and muscle denervation. These alterations result in a decreased contraction force and resistance.

In conclusion, women with pelvic floor dysfunction have anatomical muscle defects, disturbance of muscle function, and cellular changes involving muscle cells and nerve fibers. However, most of the available data were collected from patients with POP, and the risk of selection bias should not be overlooked.

## Data Availability

No new data were created or analyzed in this study.

## Supplementary Materials

The following supporting information can be downloaded at: https://www.mdpi.com/article/10.3390/muscles4010009/s1, Table S1. Demographic and clinical characteristics; Table S2. Methodological characteristics; Table S3. Studies’ quality assessment according to Newcastle–Ottawa Scale (7). Figure S1. Graphical representation of Newcastle–Ottawa Scale assessment for case-control studies. Figure S2. Graphical representation of Newcastle–Ottawa Scale assessment for cohort studies.

## Abbreviations

PFD	Pelvic floor dysfunction
PRISMA	Preferred Reporting Items for Systematic Reviews and Meta-Analyses
PROSPERO	International Prospective Register of Systematic Reviews
NOS	Newcastle–Ottawa Scale
POP	Pelvic organ prolapse
ArfGAP3	Adenosine diphosphate ribosylation factor GTPase activating protein 3
PGP	Protein gene product
SMCs	Smooth muscle cells
PDGF	Platelet-derived Growth Factor
α-SMA	α- Smooth muscle actin
LAM	*Levator ani* muscle
OASI	Obstetric anal sphincter injury
MRI	Magnetic Resonance Imaging
RR	Relative risk
SUI	Stress urinary incontinence
FI	Fecal incontinence
LAD	*Levator ani* defect
AP	Anteroposterior diameter
LR	Coronal diameter
HA	Hiatal area
LAW	Levator attachment width
PVM	Pubovisceral muscle
PFM	Pelvic floor muscle

## References

[1] Nygaard I. (2008). Prevalence of Symptomatic Pelvic Floor Disorders in US Women. JAMA.

[2] Islam R.M., Oldroyd J., Rana J., Romero L., Karim M.N. (2019). Prevalence of symptomatic pelvic floor disorders in community-dwelling women in low and middle-income countries: A systematic review and meta-analysis. Int. Urogynecol. J..

[3] Wu J.M., Vaughan C.P., Goode P.S., Redden D.T., Burgio K.L., Richter H.E., Markland A.D. (2014). Prevalence and trends of symptomatic pelvic floor disorders in U.S. women. Obstet. Gynecol..

[4] Frawley H., Shelly B., Morin M., Bernard S., Kari B., Digesu G.A., Dickinson T., Goonewardene S., McClurg D., Rahnama’i M.S. (2021). An International Continence Society (ICS) report on the terminology for pelvic floor muscle assessment. Neurourol. Urodyn..

[5] Salvador J.C., Coutinho M.P., Venâncio J.M., Viamonte B. (2019). Dynamic magnetic resonance imaging of the female pelvic floor—A pictorial review. Insights Imaging.

[6] Moher D., Shamseer L., Clarke M., Ghersi D., Liberati A., Petticrew M., Shekelle P., Stewart L.A., Prisma-P Group (2015). Preferred reporting items for systematic review and meta-analysis protocols (PRISMA-P) 2015 statement. Syst. Rev..

[7] Wells G.A., Shea B., O’Connell D., Peterson J., Welch V., Losos M., Tugwell P. (2011). The Newcastle-Ottawa Scale (NOS) for Assessing the Quality of Nonrandomised Studies in Meta-Analyses.

[8] Sun Y., Li B., Lu D., Liu C., Hong S., Hong L. (2021). Expression of ArfGAP3 in Vaginal Anterior Wall of Patients with Pelvic Floor Organ Prolapse in Pelvic Organ Prolapse and Non–Pelvic Organ Prolapse Patients. Female Pelvic Med. Reconstr. Surg..

[9] Busacchi P., Perri T., Paradisi R., Oliverio C., Santini D., Guerrini S., Barbara G., Stanghellini V., Corinaldesi R., De Giorgio R. (2004). Abnormalities of somatic peptide-containing nerves supplying the pelvic floor of women with genitourinary prolapse and stress urinary incontinence. Urology.

[10] North C.E., Creighton S.M., Smith A.R.B. (2013). A comparison of genital sensory and motor innervation in women with pelvic organ prolapse and normal controls including a pilot study on the effect of vaginal prolapse surgery on genital sensation: A prospective study. BJOG Int. J. Obstet. Gynaecol..

[11] Altman D., Zhang A., Falconer C. (2006). Innervation of the rectovaginal wall in patients with rectocele compared to healthy controls. Neurourol. Urodyn..

[12] Vetuschi A., D’Alfonso A., Sferra R., Zanelli D., Pompili S., Patacchiola F., Gaudio E., Carta G. (2016). Changes in muscularis propria of anterior vaginal wall in women with pelvic organ prolapse. Eur. J. Histochem..

[13] Heilbrun M.E., Nygaard I.E., Lockhart M.E., Richter H.E., Brown M.B., Kenton K.S., Rahn D.D., Thomas J.V., Weidner A.C., Nager C.W. (2010). Correlation between levator ani muscle injuries on magnetic resonance imaging and fecal incontinence, pelvic organ prolapse, and urinary incontinence in primiparous women. Am. J. Obstet. Gynecol.

[14] Van Delft K., Sultan A.H., Thakar R., Schwertner-Tiepelmann N., Kluivers K. (2014). The relationship between postpartum levator ani muscle avulsion and signs and symptoms of pelvic floor dysfunction. BJOG Int. J. Obstet. Gynaecol..

[15] Dietz H.P. (2007). Quantification of major morphological abnormalities of the levator ani. Ultrasound Obstet. Gynecol..

[16] Volløyhaug I., Taithongchai A., Van Gruting I., Sultan A., Thakar R. (2019). Levator ani muscle morphology and function in women with obstetric anal sphincter injury. Ultrasound Obstet. Gynecol..

[17] Lammers K., Fütterer J.J., Inthout J., Prokop M., Vierhout M.E., Kluivers K.B. (2013). Correlating signs and symptoms with pubovisceral muscle avulsions on magnetic resonance imaging. Am. J. Obstet. Gynecol..

[18] Dietz H., Simpson J. (2008). Levator trauma is associated with pelvic organ prolapse. BJOG Int. J. Obstet. Gynaecol..

[19] DeLancey J.O.L., Kearney R., Chou Q., Speights S., Binno S. (2003). The appearance of levator ani muscle abnormalities in magnetic resonance images after vaginal delivery. Obstet. Gynecol..

[20] Oberwalder M., Dinnewitzer A., Baig M.K., Thaler K., Cotman K., Nogueras J.J., Weiss E.G., Efron J., Vernava A.M., Wexner S.D. (2004). The Association between Late-Onset Fecal Incontinence and Obstetric Anal Sphincter Defects. Arch. Surg..

[21] Dietz H.P., Kirby A., Shek K.L., Bedwell P.J. (2009). Does avulsion of the puborectalis muscle affect bladder function?. Int. Urogynecol. J..

[22] Handa V.L., Blomquist J.L., Roem J., Muñoz A., Dietz H.P. (2019). Pelvic Floor Disorders After Obstetric Avulsion of the Levator Ani Muscle. Female Pelvic Med. Reconstr. Surg..

[23] Tunn R., Goldammer K., Neymeyer J., Gauruder-Burmester A., Hamm B., Beyersdorff D. (2006). MRI morphology of the levator ani muscle, endopelvic fascia, and urethra in women with stress urinary incontinence. Eur. J. Obstet. Gynecol. Reprod. Biol..

[24] Luo Y., Yang L., Lin N., Fan Z. (2021). Comparison of translabial three-dimensional ultrasonography and magnetic resonance imaging for the grading of levator ani defects. Medicine.

[25] Murad-Regadas S.M., Fernandes G.O.D.S., Regadas F.S.P., Rodrigues L.V., Pereira J.D.J.R., Dealcanfreitas I.D., Regadas Filho F.S.P. (2014). Assessment of pubovisceral muscle defects and levator hiatal dimensions in women with faecal incontinence after vaginal delivery: Is there a correlation with severity of symptoms?. Color. Dis..

[26] Hsu Y., Chen L., Huebner M., Ashton-Miller J.A., DeLancey J.O.L. (2006). Quantification of levator ani cross-sectional area differences between women with and those without prolapse. Obstet. Gynecol..

[27] Del Vescovo R., Piccolo C.L., Vecchia NDella Giurazza F., Cazzato R.L., Grasso R.F., Zobel B.B. (2014). MRI role in morphological and functional assessment of the levator ani muscle: Use in patients affected by stress urinary incontinence (SUI) before and after pelvic floor rehabilitation. Eur. J. Radiol..

[28] Castro-Pardiñas M.A., Torres-Lacomba M., Navarro-Brazález B. (2017). Muscle function of the pelvic floor in healthy and puerperal women and with pelvic floor dysfunction. Actas Urol. Españ..

[29] Morin M., Bourbonnais D., Gravel D., Dumoulin C., Lemieux M.C. (2004). Pelvic floor muscle function in continent and stress urinary incontinent women using dynamometric measurements. Neurourol. Urodyn..

[30] DeLancey J.O.L., Morgan D.M., Fenner D.E., Kearney R., Guire K., Miller J.M., Hussain H., Umek W., Hsu Y., Ashton-Miller J.A. (2007). Comparison of Levator Ani Muscle Defects and Function in Women With and Without Pelvic Organ Prolapse. Obstet. Gynecol..

[31] Tosun G., Peker N., Tosun Ö.Ç., Yeniel Ö.A., Ergenoğlu A.M., Elvan A., Yıldırım M. (2019). Pelvic floor muscle function and symptoms of dysfunctions in midwifes and nurses of reproductive age with and without pelvic floor dysfunction. Taiwan J. Obstet. Gynecol..

[32] Borello-france D.F., Handa V.L., Brown M.B., Goode P., Kreder K., Scheufele L.L., Weber A.M., Pelvic Floor Disorders Network (2007). Pelvic-Floor Muscle Function in Women with Pelvic Organ Prolapse. Phys. Ther..

[33] Handa V.L., Roem J., Blomquist J.L., Dietz H.P., Muñoz A. (2019). Pelvic organ prolapse as a function of levator ani avulsion, hiatus size, and strength. Am. J. Obstet. Gynecol..

[34] Davidson M.J., Nielsen P.M.F., Taberner A.J., Kruger J.A. (2020). Change in levator ani muscle stiffness and active force during pregnancy and post-partum. Int. Urogynecol. J..

[35] Hilde G., Stær-Jensen J., Siafarikas F., Engh M.E., Brækken I.H., Bo K. (2013). Impact of childbirth and mode of delivery on vaginal resting pressure and on pelvic floor muscle strength and endurance. Am. J. Obstet. Gynecol..

[36] Chantarasorn V., Shek K.L., Dietz H.P. (2011). Sonographic detection of puborectalis muscle avulsion is not associated with anal incontinence. Aust. N. Z. J. Obstet. Gynaecol..

[37] Lewicky-Gaupp C., Brincat C., Yousuf A., Patel D.A., Delancey J.O.L., Fenner D.E. (2010). Fecal incontinence in older women: Are levator ani defects a factor?. Am. J. Obstet. Gynecol..

[38] Muro S., Moue S., Akita K. (2024). Twisted orientation of the muscle bundles in the levator ani functional parts in women: Implications for pelvic floor support mechanism. J. Anat..

[39] Kato M.K., Muro S., Kato T., Miyasaka N., Akita K. (2020). Spatial distribution of smooth muscle tissue in the female pelvic floor and surrounding the urethra and vagina. Anat. Sci. Int..

[40] Nyangoh Timoh K., Moszkowicz D., Zaitouna M., Lebacle C., Martinovic J., Diallo D., Creze M., Lavoue V., Darai E., Benoit G. (2018). Detailed muscular structure and neural control anatomy of the levator ani muscle: A study based on female human fetuses. Am. J. Obstet. Gynecol..

