# Supplementary material for: The Muscle Cells in Pelvic Floor Dysfunctions: Systematic Review
Source: Muscles. 2025 Mar 18;4(1):9. doi: 10.3390/muscles4010009 (PMC12121313; doi:10.3390/muscles4010009)

Table S1. Demographic and clinical characteristics.

Author, Date (yyyy) [R]	*N*	Mean Age (y)	Study Groups`	Pelvic floor dysfunction type
Muscle function and pelvic floor dysfunction Studies				
Morin et al., 2004 [29]	89	31,9 vs 36,1	Continent vs SUI	SUI
Borello-France et al., 2007 [32]	317	61,6	POP	POP
DeLancey et al., 2007 [30]	186	56,4 vs 56,6	POP vs non POP	POP
Lewicky-Gaupp et al., 2010 [37]	26	28,7 vs 71,6 vs 71,6	YC vs OC vs OI	FI
Chantarasorn et al., 2011 [36]	397	54	Lower urinary tract symptoms and pelvic floor dysfunction	FI
Hilde et al., 2013 [35]	277	28,7	Pregnant nulliparous UI vs non UI	UI
Castro-Pardiñas et al., 2016 [28]	177	38,5 vs 33,9 vs 48	Healthy vs postpartum vs PFD	SUI, UU, FI, POP
Tosun et al., 2019 [31]	82	35,7 vs 41,2	Non PFD vs PFD	UI, POP, FI, PP, SD
Handa et al., 2019 [33]	432	43	Vaginally parous women with POP vs without POP	POP
Davidson et al., 2020 [34]	59	31,9	Singleton low-risk pregnancy nulliparous	Bladder, bowel, prolapse symptoms, SD
Structural pelvic floor muscle changes and pelvic floor dysfunction Studies				
DeLancey et al., 2003 [30]	240	29,2 vs 29,8 vs 30	Nullipara vs primiparous continent vs primiparous incontinent	SUI
Oberwalder et al., 2004 [20]	124	61,8 vs 68**	Late onset FI after vaginal delivery with vs without sphincter defects	FI
Hsu et al., 2006 [26]	60	-	POP vs non POP	POP
Tunn et al., 2006 [23]	54	52,1	SUI	SUI
Dietz, 2007 [15]	262	54*	UI and/or POP and/or recurrent urinary tract infections	UI, POP, cystocele, rectocele
Dietz et al., 2008 [18]	781	53	POP	POP
Dietz et al., 2009 [21]	425	55	Bladder dysfunction	Bladder dysfunction, POP
Heilbrun et al., 2010 [13]	206	27,8	Primiparous vaginal delivery (VD) 3rd or 4th degree anal sphincter	POP, FI, UI

			tear vs VD without anal sphincter tear vs cesarean delivery	
Lammers et al., 2013 [17]	189	55 vs 59 vs 55	POP or pelvic floor dysfunction symptoms with no pubovisceral avulsion vs minor avulsion vs major avulsion	POP and other pelvic floor dysfunction symptoms
Van Delft et al., 2014 [14]	188	30,7	Primigravida no LAM avulsion vs minor LAM avulsion vs major LAM avulsion	POP, FI, UI, SD
Del Vescovo et al., 2014 [27]	22	53,7	SUI patients before and after perineal rehabilitation	SUI
Murad-Regadas et al., 2014 [25]	69	61 vs 28	FI with vaginal delivery vs nulliparous asymptomatic	FI, UI
Handa et al., 2019 [22]	453	45,9 vs 42,9	Vaginal parous women with LAM avulsion vs vaginal parous women without LAM avulsion	POP, other pelvic floor disorders
Volløyhaug et al., 2019 [16]	248	31,5	LAM injury vs LAM intact	FI, UI
Luo et al., 2020 [24]	44	28,6 vs 29	Intact LAM vs Unilateral LAD	SUI, POP
Cellular pelvic floor muscle alterations and pelvic floor dysfunction Studies				
Busacchi et al., 2004 [9]	40	64,8 vs 61,6	POP +/- SUI vs non POP and no SUI	SUI, POP
Altman et al., 2006 [11]	32	61,4 vs 57,3	Rectocele vs without rectocele	POP, anorectal symptoms
North et al., 2013 [10]	30	57 vs 26	POP vs healthy	POP
Vetuschi et al., 2016 [12]	24	55 vs 55	POP vs non POP	POP
Sun et al., 2021 [8]	59	63 vs 62	POP vs Non POP	POP

[*] mean age of the subgroup with avulsion injuries; [**] median age at FI onset

SUI, stress urinary incontinence; UU, urinary urgency; FI, fecal incontinence; POP, pelvic organ prolapse; PFD, pelvic floor dysfunction; PP, pelvic pain; SD, sexual dysfunction; YC, young continent; OC, older continent; OI, older incontinent

Table S2. Summary of the main objectives, parameters and tools studied.

Author, Date (yyyy) [R]	*N*	Primary Outcome	Measured Parameters	Methods
		Muscle function and pelvic floor dysfunction Studies		
Morin et al., 2004 [29]	89	Pelvic floor muscle function	Passive force, rate of force, number of contractions, absolute endurance	Vaginal dynamometry
Borello-France et al., 2007 [32]	317	Pelvic floor muscle function	Squeeze pressure, contraction duration, vertical displacement	Intravaginal palpation (Brink scale)
DeLancey et al., 2007 [30]	186	Pelvic floor muscle function and LAM defects	Vaginal closure force at rest and maximal contraction<br>POP<br>LAM avulsion<br>Pelvic floor dysfunction symptoms	Instrumented vaginal speculum<br>POP-Q<br>MRI<br>Pelvic floor dysfunction questionnaire
Lewicky-Gaupp et al., 2010 [37]	26	Pelvic floor muscle structure<br>Pelvic floor muscle function (second outcome)	Levator ani rest force, levator ani maximal contraction	Instrumented speculum exam (newtons)
Chantarasorn et al., 2011 [36]	397	FI and puborectalis muscle avulsion and levator hiatus distensibility	FI<br>Muscle volumes at rest, maximum contraction and valsalva<br>Puborectalis muscle avulsion, contractility and distensibility<br>Hiatal area<br>Hiatal dimension at rest, maximum contraction and valsalva	4D pelvic floor ultrasound<br>Digital palpation (MOS)<br>POP-Q<br>FI questionnaire
Hilde et al., 2013 [35]	277	Pelvic floor muscle function	Vaginal resting pressure, pelvic floor muscle	Vaginal manometry

			strength, pelvic floor muscle endurance	
Castro-Pardiñas et al., 2016 [28]	177	Pelvic floor muscle function	Tone, strength, resistance, neuromuscular activity	Intravaginal palpation (Levator ani test)<br>Vaginal manometry<br>Vaginal dynamometry<br>Surface eletromiography
Handa et al., 2019 [33]	432	Pelvic muscle strength, LAM avulsion and levator hiatus area	Pelvic floor muscle strength<br>POP<br>Levator avulsion<br>Levator hiatus area	Peritron perineometer<br>3D transperineal ultrasound<br>Tomographic ultrasound imaging<br>POP-Q
Tosun et al., 2019 [31]	82	Pelvic floor muscle function	Power, endurance, number of repetitions, number of fast contractions, every contraction timed	Intravaginal palpation (PERFECT scheme)
Davidson et al., 2020 [34]	59	LAM stiffness and active force	Active force<br>Muscle stiffness<br>Hiatal dimensions<br>LAM avulsion<br>Pelvic floor dysfunction symptoms	Intravaginal palpation (MOS)<br>Elastometry<br>3D/4D transperineal ultrasound<br>Tomographic ultrasound imaging<br>Australian Pelvic Floor Questionnaire (APFQ)
Structural pelvic floor muscle changes and pelvic floor dysfunction Studies				
DeLancey et al., 2003 [30]	260	LAM defects	LAM defects	MRI
Oberwalder et al., 2004 [20]	124	Late onset FI and anal sphincter defects	FI<br>Anal sphincter defects<br>Squeeze and resting pressure<br>Pudendal neuropathy	Cleveland Clinic Florida Incontinence Grading Scale (CCFIS)<br>Endoanal ultrasound<br>Anal manometry<br>Pudendal nerve terminal motor latency (PNTML)
Hsu et al., 2006 [26]	60	Differences of cross-sectional area of levator ani between POP and non-POP	LAM defects<br>LAM cross-sectional area	MRI with 3D reconstruction<br>I-DEAS
Tunn et al., 2006 [23]	54	Pathomorphologic changes of LAM,	External urethral sphincter	MRI<br>SUI (Ingelman-Sundberg)<br>MRI urodynamic testing

		endopelvic fascia, urethra	muscle and configuration of urethral lumen<br>LAM intensity and avulsion<br>Central and lateral endopelvic fascia defects	POP-Q
Dietz, 2007 [15]	262	LAM abnormalities	LAM abnormalities	3D/4D pelvic floor ultrasound<br>Tomographic ultrasound imaging<br>POP-Q
Dietz et al., 2008 [18]	781	LAM defects	LAM avulsion<br>POP	4D translabial ultrasound<br>POP-Q
Dietz et al., 2009 [21]	425	Puborectalis muscle avulsion and bladder dysfunction	Muscle volumes<br>Maximal pelvic floor contraction<br>Puborectalis muscle avulsion<br>Bladder dysfunction<br>POP	3D/4D translabial ultrasound<br>POP-Q<br>Pelvic floor dysfunction symptoms questionnaire<br>Independent flowmetry<br>Multichannel urodynamic testing<br>Digital palpation (MOS)
Heilbrun et al., 2010 [13]	207	LAM injury and pelvic floor dysfunction	LAM avulsion<br>FI<br>UI	MRI<br>Medical Epidemiologic and Social Aspects of Aging (MESA)<br>FI Severity Index (FISI)<br>POP-Q
Lammers et al., 2013 [17]	189	POP, pelvic floor dysfunction symptoms, life quality and pubovisceral avulsion	FI<br>Obstrutive defecation<br>UI<br>POP<br>Pubovisceral avulsion	Urogenital Distress Inventory (UDI)<br>Incontinence Impact Questionnaire (IIQ)<br>Defecatory Distress Inventory (DDI)<br>POP-Q examination<br>MRI
Van Delft et al., 2014 [14]	188	LAM avulsion and pelvic floor muscle strength and pelvic floor dysfunction	LAM avulsion<br>Hiatus area<br>Pelvic floor muscle strength<br>FI<br>UI<br>SD	Transperineal ultrasound 3D/4D<br>Intravaginal palpation (MOS)<br>POP-Q<br>St Mark`s incontinence score<br>International Consultation Incontinence Questionnaire Short Form (ICIQ-SF)<br>ICIQ-VS

Del Vescovo et al., 2014 [27]	22	Effectiveness of rehabilitation treatment applying MRI technique for morphologic and functional assessment of LAM	Urodynamic evaluation<br>Tropism of the ventral structure of the LAM<br>Thickness of each side of the LAM	Urodynamic evaluation<br>MRI with 3D reconstruction<br>ICIQ-UI questionnaire
Murad-Regadas et al., 2014 [25]	69	Pubovisceral muscle defects (PVM) and levator hiatal dimension and fecal incontinence symptoms severity	FI<br>UI<br>PVM defects<br>Levator hiatus<br>Sphincter defects	Cleveland Clinic Florida Incontinence Scoring System<br>3D endovaginal and endorectal ultrasonography
Handa et al., 2019 [22]	453	POP and other PFD incidence difference between parous women with and without LAM avulsion	LAM avulsion<br>POP<br>UI<br>Overactive bladder<br>FI	3D transperineal ultrasound<br>POP-Q<br>Epidemiology of Prolapse and Incontinence Questionnaire
Volløyhaug et al., 2019 [16]	248	LAM morphology and function	FI<br>UI<br>LAM injury<br>Levator hiatus	St Mark`s incontinence score<br>International consultation on incontinence modular questionnaire for urinary incontinence – short form<br>3D/4D transperineal ultrasound<br>Tomographic ultrasound<br>Modified oxford scale
Luo et al., 2020 [24]	44	Unilateral high grade tears affects LAM morphology and overdistention	LAM tears<br>Levator attachment width<br>Hiatal area<br>LAM distensibility<br>UI, FI, POP	Translabial ultrasonography<br>Tomographic ultrasound<br>MRI with 3D reconstruction<br>UI, FI, POP questionnaires<br>POP-Q examination
Cellular pelvic floor muscle alterations and pelvic floor dysfunction Studies				
Busacchi et al., 2004 [9]	40	POP and peripheral nerve abnormalities	Immunolabeled nerves quantification and distribution<br>POP<br>SUI	Periurethral and perirectal muscles biopsy<br>Immunohistochemistry (antibodies protein S-100, neuron-specific enolase, neuropeptides)<br>Urodynamic investigations

				Pelvic floor muscles electromyography<br>POP-Q examination
Altman et al., 2006 [11]	32	Rectovaginal innervation	Nerve fibre density<br>Rectocele size<br>POP<br>Anorectal symptoms	Rectovaginal wall biopsy<br>Immunofluorescence staining and quantification (PGP-9.5 antibodies)<br>POP examination<br>Defecoperitoneography<br>Anorectal symptoms questionnaire
North et al., 2013 [10]	30	Genital sensory and motor enervation and POP	POP<br>Genital vibration threshold before and after surgery<br>Percentage of polyphasic potentials (marker of previous motor denervation)	POP-Q examination<br>Sensory and concentric needle electromyography (CNEMG) testing
Vetuschi et al., 2016 [12]	24	*Muscularis propria* changes of anterior vaginal wall and POP	Smooth muscle fibres<br>Extracellular matrix<br>POP	Anterior vaginal wall biopsy<br>Immunohistochemical analysis (polyclonal antibodies to collagen I, collagen III, α-smooth muscle actin, platelet derived growth factor, matrix metalloproteinase 3, tissue inhibitors for metalloproteinases, caspase 3)<br>Immunofluorescence staining (collagen I, III, platelet derived growth factor)
Sun et al., 2021 [8]	59	Molecular expression of vaginal anterior wall	Adenosine diphosphate ribosylation factor GTPase-activating protein 3	Vaginal hysterectomy<br>Immunohistochemical staining<br>Western blotting<br>Quantitative real-time fluorescence polymerase chain reaction

Table S3. Studies quality assessment according NewCastle-Ottawa Scale(7).

**Study**	Selection (4)				Comparability (2)	Outcome/Exposure (3)		
Muscle function and pelvic floor dysfunction Studies								
Morin et al., 2004								
Borello-France et al., 2007								
DeLancey et al., 2007								
Lewicky-Gaupp et al., 2010								
Chantarasorn et al., 2011								
Hilde et al., 2013								
Castro-Pardiñas et al., 2016								
Tosun et al., 2019								
Handa et al., 2019								
Davidson et al., 2020								
Structural pelvic floor muscle changes and pelvic floor dysfunction Studies								
DeLancey et al., 2003								
Oberwalder et al., 2004								
Hsu et al., 2006								
Tunn et al., 2006								
Dietz, 2007								
Dietz et al., 2008								
Dietz et al., 2009								
Heilbrun et al., 2010								
Lammers et al., 2013								
Van Delft et al., 2014								
Del Vescovo et al., 2014								
Murad-Regadas et al., 2014								

Handa et al., 2019								
Volløyhaug et al., 2019								
Luo et al., 2020,								
Cellular pelvic floor muscle alterations and pelvic floor dysfunction Studies								
Busacchi et al., 2004 [9]								
Altman et al., 2006								
North et al., 2013								
Vetuschi et al., 2016								
Sun et al., 2021								

Figure S1. Graphical representation of Newcastle Ottawa Scale assessment for case-control studies.

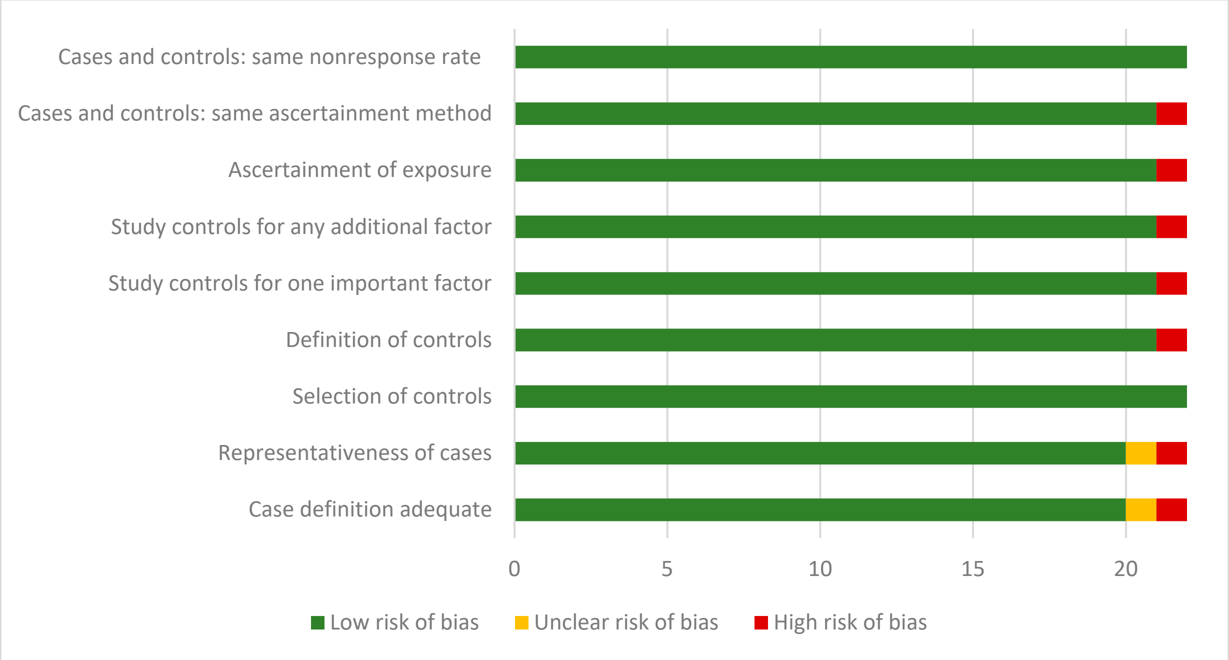

Figure S2. Graphical representation of Newcastle Ottawa Scale assessment for cohort studies.

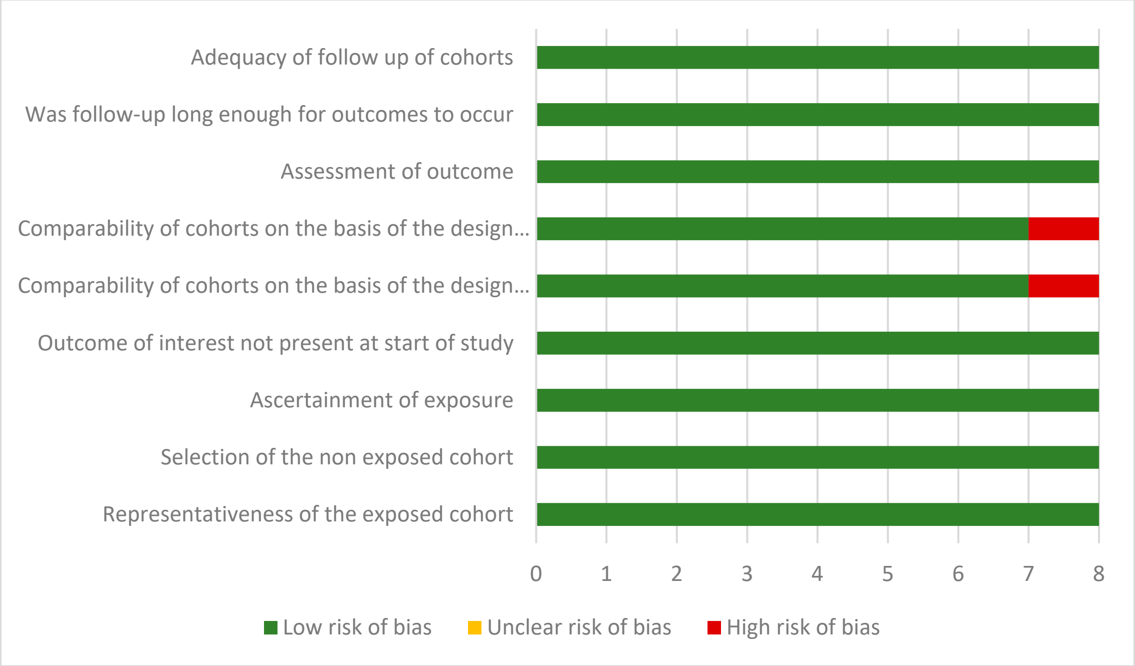

Supplement: Supplementary file 1 [file muscles-04-00009-s001.zip › muscles-3315652-supplementary.pdf]
